# Evaluation of three cryoprotectants used with bovine milk affected with *Mycoplasma bovis* in different freezing conditions

**DOI:** 10.1186/s13104-018-3325-6

**Published:** 2018-04-02

**Authors:** Abd Al-Bar Al-Farha, Manouchehr Khazandi, Farhid Hemmatzadeh, Razi Jozani, Rick Tearle, Andrew Hoare, Kiro Petrovski

**Affiliations:** 1 School of Animal and Veterinary Sciences, The University of Adelaide, Roseworthy, Adelaide, SA 5371 Australia; 2 Mosul Technical Institute, Northern Technical University, Technical Foundation, Mosul, Iraq; 3 Australian Centre for Antimicrobial Resistance Ecology, The University of Adelaide, Roseworthy, Adelaide, SA 5000 Australia; 4 Davies Centre, School of Animal and Veterinary Sciences, The University of Adelaide, Roseworthy, Adelaide, SA 5371 Australia; 5 Department of Veterinary Clinical Sciences, University of Tabriz, Tabriz, Iran; 6 South East Vets, Mt Gambier, SA 5290 Australia

**Keywords:** *Mycoplasma bovis*, Mastitis, Cryopreservation, Glycerol, DMSO, Gelatin

## Abstract

**Objectives:**

Currently, there is no consensus protocols regarding the combination of glycerol (GLY), gelatin or foetal bovine serum (FBS) with dimethyl sulphoxide (DMSO) as cryoprotectants for *Mycoplasma bovis* in bovine milk samples. This study aimed to compare different cryopreservation compounds and storage temperatures for *M. bovis*.

**Results:**

There were significant differences in the survival of *M. bovis* on different media. Differences were also observed between different storage conditions. All additives improved the survival of *M. bovis* in comparison to control (CON). The combination of GLY and DMSO was shown to be significantly different to CON with 57.1% (95% CI = 21.43–133.34) and 19.1% (95% CI = 11.73–60.27), respectively at week 16, and its use should be encouraged as a cryoprotectant for *M. bovis* at − 20 and − 80 °C. GEL/DMSO showed the highest survival rate for *M. bovis* with 57.14% (95% CI = 21.43–133.34) at 4 °C in comparison with CON 14.29% (95% CI = 9.60–50.39). FBS/DMSO showed the highest survival rate for the short-term preservation similarly to other additives. The evaluated cryopreservative compounds would improve survivability of *M. bovis* in milk for both transport and long-term storage. Hence, it is recommended to use the mentioned methods for routine transportation or storage purposes for suspicious *M. bovis* milk samples.

## Introduction

*Mycoplasma bovis* mastitis is increasingly generating considerable interest in the bovine dairy industry. The current method for isolation of mycoplasmas is using specific mycoplasma culture media. For this purpose, milk samples are sent as frozen or fresh to diagnostic laboratories. An important factor for successful bacterial isolation is to keep *Mycoplasma* organisms viable for growth. Consequently, appropriate sample handling and storage is the main key for diagnosis or research purposes. Due to the lack of cell wall, viability of *M. bovis* under freeze-thaw conditions is a significant challenge in short and long-term preservation. Previous studies have focused on different animal sources of mycoplasmas rather than bovine milk [1, 2]. For bovine milk, standard protocols for prolonged storage of non-*Mycoplasma* mastitis pathogens have been proposed [3–5]. However, these protocols are not applicable to *M. bovis* due to the structural variation between these bacteria and conventional mastitis pathogens. Furthermore, farmers commonly freeze collected milk samples for submission to the diagnostic labs as part of their mastitis management program or if mastitis is a perceived problem on their farm (e.g. increase in incidence of mastitis or treatment failure). Preserving the collected milk is the most important step for bacterial isolation/detection in both bacteriological and molecular methods. A cornerstone for prospective microbiological studies is successful culture of *Mycoplasma.* For routine culture of *Mycoplasma* mastitis, the use of fresh milk samples has been recommended [6]. However, preserved milk samples should be considered. Thus, appropriate storage conditions are required to obtain maximal survival of *M. bovis*. A dearth of knowledge regarding appropriate storage of milk samples, both for farmers and researchers, is evident. This study evaluated survival of *M. bovis* in bovine milk following various storage times under three different temperature storage conditions (4 , − 20 and − 80 °C) using milk only as a control (CON) or three different storage media [milk supplemented with dimethyl sulphoxide (DMSO) and foetal bovine serum (FBS), gelatin (GEL) or glycerol (GLY)].

## Main text

### Methods

#### Milk samples

As part of a previous study, milk samples were collected aseptically from 288 cows at individual cow-level from a single commercial dairy farm near Mount Gambier in South Australia [7]. All samples were subjected to cryopreservation, culture and PCR. In this study, twenty-one positive samples for *M. bovis* were selected based on positive culture and PCR results.

#### *Bovis* culture

Milk samples were subjected to *Mycoplasma* culture using *Mycoplasma* selective media (Oxoid, Sydney, Australia) according to the manufacturer’s instructions. *Mycoplasma* colonies were counted using a stereomicroscope at 10× magnification after 7–14 days. Cultures were considered positive when a minimum of one *M*. *bovis* colony was recorded [8]. At the moment of counting the person who carried out the procedure was not aware of the group allocation. The initial concentration of the organisms in each milk sample was calculated at week 0 for all samples.

#### Identification of *M. bovis* by PCR

DNA was extracted directly from milk using QIAmp DNA extraction kit (Qiagen, Germany) according to the manufacturer’s instructions. Specific 16S rRNA primers designed for *M. bovis* (442 bp), composed of Mbov-F: 5′-CCAGCTCACCCTTATACATGAGCGC-3′ and Mbov-R: 5′-TGACTCACCAATTAGACCGACTATTTCACC-3′. Amplifications were carried out in 25 µL containing 0.25 µL Taq DNA polymerase, 5 µL of 5× reaction buffer (Bioline, UK), 1 µL (0.5 µM) of each forward and reverse primers, 1 µL (approximately 20 ng) of template, and 16.75 µL of DEPC-treated water. Amplifications were performed for 35 PCR cycles conditions using T100™ Thermal Cycler (Biorad thermocycler, Australia), and consisted of pre-heating activation for 5 min at 95 °C, denaturation at 95 °C for 30 s, annealing at 60 °C and primer extension at 72 °C for 45 s. The final extension step was performed at 72 °C for 10 min. The PCR products were analysed by 1.5% agarose gel electrophoresis and visualised by staining with Gel Red.

#### Evaluating storage-recovery of *M. bovis*

The following storage media were selected for this study: (a) milk supplemented with 40% FBS and 10% DMSO (treatment group FBS), (b) milk supplemented with 40% GEL (conc. 150 g/L) and 10% DMSO (treatment group GEL), (c) milk supplemented with 40% GLY and 10% DMSO (treatment group GLY), and (d) milk alone (treatment group CON). For each preparation, 8 mL of each *M. bovis* positive milk sample were added into 8 mL of each storage medium, and CON. Each of the diluted samples was aliquoted into 15 Eppendorf tubes (1 mL each) at the same day of collection. For each storage medium, five tubes of aliquots was stored at 4, − 20 or − 80 °C. There were therefore 21 samples × 4 storage media × 3 storage conditions × 5 time points, totalling 1260 combinations. At each particular time point, one aliquot from each storage media was thawed and cultured onto *Mycoplasma* selective media as described above.

#### Statistical analysis

The data were binomially distributed (0 = no recovery; 1 = recovery). Hence, a generalised linear model using (R version 3.1.1, R Development Core Team, New Zealand) package was run for the dataset. The predicted survival was estimated accounting for the fixed effect of storage media (FBS, GEL, GLY, or CON), time (weeks), storage condition (4, − 20 or − 80 °C), and their three-way interaction. Means of survival as rate, 95% confidence intervals, and differences between means were obtained, and are used in the comparison between storage media, time and storage conditions. Survival analysis per treatment group was also carried out and results are presented as figures.

Analysis of variance (ANOVA) using MIXED of the SAS of the number of colony-forming units (CFUs) per plate at each time point was estimated accounting for the fixed effect of storage media (FBS, GEL, GLY, or CON), time (weeks), storage condition (4, − 20 or − 80 °C), and their three-way interaction. Means of CFU, standard errors and were obtained, and are used in the comparison between storage media, time and storage conditions.

### Results

**Fig. 1:**
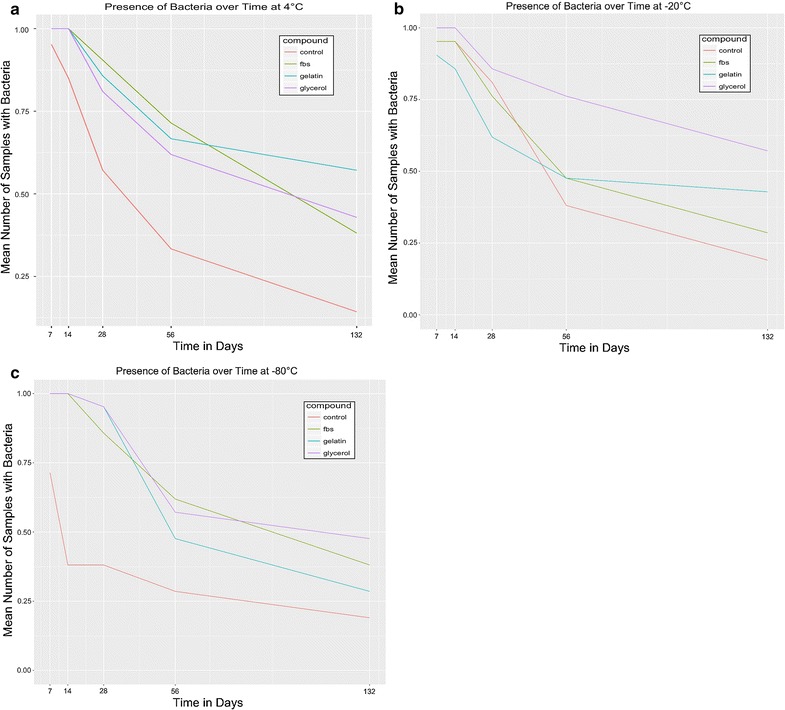
Viability percentage of *Mycoplasma bovis* after storage at domestic fridge 4 °C (**a**), domestic freezer − 20 °C (**b**), or laboratory freezer − 80 °C (**c**) assessed in milk or milk supplemented with Foetal Bovine Serum (40% v-v) + Dimethyl Sulphoxide (10% v-v), Gelatin (40% v-v) + DMSO (10% v-v) or Glycerol (40% v-v) + DMSO (10% v-v)

The viability of *M. bovis* for the 21 milk samples after storage at different temperature conditions and milk alone or in combination with three different cryoprotectants (treatment groups FBS, GEL, GLY or CON) are shown in Fig. 1. A significant differences in survival rate of *M. bovis* were detected between different cryoprotectants and temperature conditions. In general, all additives improved the survival rate of *M. bovis* in comparison with CON. The highest survival rate for *M. bovis* isolates was observed at − 80 °C followed by − 20 and 4 °C. For the long term preservation, GLY was the most effective cryoprotectant; at − 20 and − 80 °C the survival rate was 57.1% (95% CI = 21.43–133.34) and 47.6% (95% CI = 20–116.04), respectively, in comparison with CON 19.1% (95% CI = 11.73–60.27) in week 16. GEL showed highest *M. bovis* survival rate at 4 °C with 57.1% (95% CI = 21.4–133.34) in comparison with CON 14.3% (95% CI = 9.6–50.39) in week 16. FBS showed the highest survival rate for the short-term preservation, similarly to other additives. However, contrary to survivability rates, no significant differences were observed in the CFUs among the survived isolates Table 1. Agarose gel electrophoresis for specific *M. bovis* PCR of all 21 samples tested revealed amplicon size of 442 bp.

**Table 1:** The mean values (± SE) of viable colonies of *Mycoplasma bovis* for 21 milk samples after storage at 4, − 20, or − 80 °C in milk or milk supplemented with Foetal Bovine Serum (40% v-v) + Dimethyl Sulphoxide (10% v-v), Gelatin (40% v-v) + DMSO (10% v-v) or Glycerol (40% v-v) + DMSO (10% v-v)

Storage time (weeks)	The number of *M. bovis* (CFU/mL)											
	4 °C				− 20 °C				− 80 °C			
	CON^a^	FBS^b^	GEL^c^	GLY^d^	CON	FBS	GEL	GLY	CON	FBS	GEL	GLY
1	301.7 ± 94.5	327.6 ± 97.4	361.9 ± 100	312 ± 101	304.7 ± 93.7	342.8 ± 99.5	323.8 ± 96.3	320 ± 100.7	316.1 ± 106.9	327.6 ± 58.1	342.8 ± 100.5	320 ± 100.9
2	285.3 ± 95.8	300.9 ± 92	369.5 ± 99	281 ± 91	281.9 ± 90.6	335.2 ± 94.9	327.6 ± 91.0	308.5 ± 96.1	204.2 ± 90.8	266.6 ± 58.5	300.9 ± 100.1	350.4 ± 105.9
4	154.6 ± 23.3	194 ± 64.3	388 ± 102	300 ± 96	262.8 ± 85.9	274.2 ± 79.7	300.9 ± 110.3	297.1 ± 93.8	117.1 ± 43.8	240 ± 63.8	281.9 ± 89.1	380.9 ± 98.7
8	56.5 ± 23.8	125.7 ± 46	201.9 ± 75.3	289.5 ± 95	137.1 ± 74.9	148.5 ± 52.4	262.8 ± 110.0	335.2 ± 103.3	38 ± 20.9	91.4 ± 20.9	224.7 ± 96.8	384.7 ± 102.3
16	16.6 ± 11.4	72.3 ± 28.9	160 ± 66.6	186.6 ± 73.7	57.1 ± 34	83.8 ± 42.2	110.4 ± 52.8	251.4 ± 78.1	11.4 ± 6.1	38.1 ± 19.6	144.7 ± 83.6	266.6 ± 94.5

^a^Control (milk only); ^b^ Foetal Bovine Serum (40% v-v) + Dimethyl Sulphoxide (10% v-v); ^c^ Gelatin (40% v-v) + DMSO (10% v-v); ^d^ Glycerol (40% v-v) + DMSO (10% v-v)

### Discussion

The effect of *M. bovis* viability under freeze-thaw conditions is a significant challenge in preservation of these bacteria. Lacking of peptidoglycan cell wall in mycoplasmas makes them sensitive to formation of ice crystal during freezing/thawing processes. Sensitivity of *Mycoplasma* spp. to freezing injuries due to phospholipid membrane lipids leakage has been reported in previous studies [9]. *M. bovis* is a fastidious pathogen, and its survival during storage is often affected by both bacterial overgrowth and pH alteration of milk [10]. Intracellular and extracellular ice crystallisation play an important role in cell damage during freezing processes [9, 11]. Our results indicated maximal survival rate of isolates after short- and long-term storage in the treatment group GLY. We hypothesise that optimum survival in freezing conditions containing DMSO was a result of prevention of formation of intracellular ice crystals [12]. GLY has a similar role in cryopreservation that likely results from binding the hydrogen–hydrogen bonds of water intracellularly [13]. The bacteriostatic activity of GLY contributes to inhibition of other bacterial growth leading to improved survival of *Mycoplasma* [14]. The effect of DMSO and GLY as cryoprotectants for various kinds of microorganisms has been previously reported [15]. A lower survival was detected when a combination of FBS and DMSO were used. FBS has been used as a preservation for many types of cells either alone or in combination with DMSO. It is hypothesised that FBS protects cells from osmotic shock, in addition to the neutralising activity against toxic materials released from the damaged cell during the freezing process [15–17]. GEL has been previously used as a preservative for various bacterial species [18], and can act as coating factor to cells similar to GLY. Together, these findings demonstrate that the combination of DMSO + GLY significantly preserves the viability of *M. bovis* under different storage conditions. Our results indicated the legitimacy of using a combination of GEL + DMSO solution as well as FBS + DMSO as additives to milk samples stored at different temperatures.

In conclusion, this study revealed that milk samples supplemented with DMSO and GEL or GLY improved the survival of *M. bovis* associated with mastitis. Our cryoprotectants need to be studied for conventional mastitis pathogens. If the results are similar, addition of preservation additives used in this study can be recommended as a routine procedure for transforming or storing milk samples for different purpose processing.

## Limitations

Although there was a dramatic decrease in CFU over the period of preservation points, no significant differences were found. This may be a true effect but may also be due to small sample size. Samples used in this study were not randomly selected. They were selected by chance from the original pool of samples. Hence, one may suspect that the results may not be applicable to the external population. However, in this study each sample had similar chance of being selected for the study, as cows were sampled in non-particular order, and selection of milk samples was in non-particular order as well.

## Abbreviations

CON: control
GLY: glycerol
GEL: gelatin
FBS: foetal bovine serum
DMSO: dimethyl sulphoxide
CFU: colony forming unit
